# Tomato ripeness detection and fruit segmentation based on instance segmentation

**DOI:** 10.3389/fpls.2025.1503256

**Published:** 2025-05-02

**Authors:** Jinfan Wei, Yu Sun, Lan Luo, Lingyun Ni, Mengchao Chen, Minghui You, Ye Mu, He Gong

**Affiliations:** ^1^ College of Information Technology, Jilin Agricultural University, Changchun, China; ^2^ Jilin Province Intelligent Environmental Engineering Research Center, Changchun, China

**Keywords:** tomato instance segmentation, complex field environments, adaptive feature extraction, multi-scale features, self-attention mechanism, ACP-tomato-seg

## Abstract

In order to meet the urgent need of fruit contour information for robot precision picking in complex field environments (such as light changes, occlusion and fruit overlap, etc.), this paper proposes an improved YOLOv8s-seg method for tomato instance segmentation, named ACP-Tomato-Seg. The method proposes two innovative modules: the Adaptive and Oriented Feature Refinement module (AOFRM) and the Custom Multi-scale Pooling module (CMPRD) with Residuals and Depth. By deformable convolution and multi-directional asymmetric convolution, the AOFRM module adaptively extracts the shape and direction features of tomatoes to solve the problems of occlusion and overlap. The CMPRD module uses the pooling kernels of self-defined size to extract multi-scale features, which enhances the model’s ability to distinguish tomatoes of different sizes and maturity levels. In addition, this paper also introduces a partial self-attention module (PSA), which combines channel attention and spatial attention mechanism to capture global context information, improve the model’s ability to focus on the target region and extract details. To verify the validity of the method, a dataset of 1061 images of large and small tomatoes was constructed, covering six ripened categories of large and small tomatoes. The experimental results show that compared with the original YOLOv8s-seg model, the performance of ACP-TomatoSeg model is significantly improved. In the bounding box task, mAP50 and MAP50-95 are improved by 5.6% and 8.3%, respectively, In the mask task, mAP50 and MAP50-95 increased by 5.8% and 8.5%, respectively. Furthermore, additional validation on the public strawberry instance segmentation dataset (StrawDI_Db1) indicates that ACP-Tomato-Seg not only exhibits superior performance but also significantly outperforms existing comparative methods in key metrics. This validates its commendable generalization ability and robustness. The method showcases its superiority in tomato maturity detection and fruit segmentation, thus providing an effective approach to achieving precise picking.

## 1 Introduction

Modern agriculture is developing in the direction of automation and intelligence, in which robot automatic picking technology is a key link to improve agricultural production efficiency and reduce labor costs (Oliveira et al., 2021). Globally, Tomatoes are the second most grown vegetable crop after potatoes (Ma et al., 2023), Accurate detection of ripeness and precise segmentation of fruit are essential for efficient and precise automatic harvesting. Traditional agricultural practices, mainly rely on manual tomato picking, which is not only labor-intensive, inefficient, and difficult to meet the growing market demand.

In the early days, limited by the technical level, people tried to use simple mechanical devices for batch picking, but this way could not identify the maturity of the fruit, and could not achieve accurate picking, resulting in serious damage to the fruit and mixed quality. With the rise of digital image processing and machine learning algorithms, researchers began to explore the use of machine vision technology to give robots the ability to “recognize objects” (Wang T. et al., 2022). For a long time in the past, researchers began to explore the use of digital image processing technology and machine learning algorithms to automatically identify the maturity of various fruits and vegetables, mainly by extracting color features or texture features in the image to match the maturity of fruits and vegetables, such as: Wan et al. (2018) combined image processing technology and backpropagation neural network to obtain tomato images, extract color feature values, and use these feature values as inputs to train and verify models, so as to realize automatic detection and classification of fresh tomato maturity. Anindita Septiarini et al. (2021) extracted the color and texture features of fresh oil palm fruit clusters, and used principal component analysis (PCA) for feature selection to select the most influential features, and then used artificial neural network and backpropagation algorithm to classify the maturity of fresh oil palm fruit clusters. Although these methods have achieved some success in feature design, their limitations have become increasingly prominent. These methods rely too much on fixed color characteristics, and have insufficient adaptability to light and color changes, which is difficult to meet the challenges brought by the color diversity of fruits and vegetables and the change of environmental light. In addition, the traditional texture feature extraction method is based on artificially designed filters and feature descriptors, which has the problems of low computational efficiency and insufficient generalization ability, and it is difficult to deal with a wide variety of fruit and vegetable textures.

In recent years, with the rapid development of computer vision and artificial intelligence technologies, automated picking systems based on machine vision have gradually emerged (Tang et al., 2020). Among them, object detection technology has been widely applied to the recognition and localization of fruits and vegetables. Particularly in the field of tomato detection and maturity classification, researchers have achieved remarkable progress. For instance, JUN Guo et al. (2023). expanded the receptive field by introducing the ReplkDext structure, optimized the detection head, and adopted the ODConv module, which improved the detection accuracy (mAP50 increased by 1.3%) in densely occluded scenarios. Congyue Wang et al (2023). optimized the anchor boxes based on K-means++, combined the coordinate attention (CA) mechanism with the WIoU loss function, and achieved a high average precision of 95.2% while ensuring real - time performance (5.3 ms/frame). Appe et al. (2023). integrated the channel-spatial attention (CBAM) with the DioU-NMS strategy, raising the mAP50 of YOLOv5 to 88.1% on the Laboro Tomato dataset, effectively enhancing the recognition ability under occlusion and sudden illumination changes. (Nguyen et al., 2024) utilized the lightweight RFAConv module, achieving an mAP50 of 88.2% and a speed of 10.3 ms/frame with an extremely low parameter count (3.06M), demonstrating the potential for deployment on low-computing-power devices. The TOMATOD dataset constructed by the Tsironis team (Tsironis et al., 2020) and their fine-grained maturity classification evaluation of mainstream detection algorithms have promoted the standardization of algorithm evaluation in this field. In addition, in other fruit and vegetable recognition tasks, Gao Ang et al. (2024) aiming to improve the detection efficiency and accuracy of citrus young fruits, proposed an improved detection method based on YOLOV8n. By using a lightweight network and integrating the attention mechanism, they enhanced the model’s detection ability. Yan Liu et al. (2023) proposed a blueberry maturity detection algorithm based on the YOLOv5x algorithm, aiming to enhance the ability of picking robots to automatically recognize and pick ripe blueberries. They optimized the algorithm structure by introducing the lightweight attention mechanism Little-CBAM and the improved MobileNetv3. When deployed on a picking robot, it can operate in real - time at a speed of 47 frames per second, showing good practicality and accuracy. However, although these studies based on object detection technology have achieved remarkable results in terms of localization accuracy and classification accuracy, their output results are essentially the bounding boxes of objects. Such rectangular boxes can only provide approximate position and category information of the objects, and it is impossible to obtain precise pixel-level contours. In scenarios that require fine-grained operations, such as robot picking, accurate fruit contours are crucial for planning grasping postures, calculating grasping points, and avoiding stem and leaf occlusions. The bounding box information has inherent limitations in this regard.

In contrast, instance segmentation technology can not only detect objects but also accurately segment the pixel-level masks of each object, providing complete contour information, which offers key technical support for downstream tasks such as precise grasping and path planning of robots. For example, Huijun Zhang et al. (2023) developed a binocular apple positioning method based on Mask R-CNN, which is used to improve the positioning performance of apple robot picking. Binocular cameras are used to capture apple images, and then Mask R-CNN is applied for instance segmentation. The 3D coordinates of Apple are calculated by using template matching and stereoscopic matching techniques. The results show that this method has excellent performance in positioning accuracy and consistency. Aiming at the occlusion problem in the visual recognition system of litchi picking robot, Yuanhong Li et al. (2023) proposed a picking location prediction method based on litchi phenotype characteristics, used edge computing and gradient direction distribution to calculate fruit normal vector, and proposed a one-stage instance segmentation network (LP3Net) based on feature prototype. The experimental results showed that, based on the phenotypic characteristics of litchi, LP3Net achieved an average localization accuracy of up to 82%, which significantly improved the localization accuracy of litchi cluster picking points. Cheng Chen et al. (2020) proposed an instance segmentation method based on monmesh RGB camera for the accurate location of sweet pepper fruit. A deep convolutional neural network (CNN) was designed to output binary segmentation maps and embedded feature maps in a multi-task framework, and the mean shift clustering and contour discovery algorithms were used to achieve instance segmentation and location. The method was validated on a public bell pepper dataset with competitive results. Isaac Pérez-Borrero et al. (2020) proposed a deep learn-based segmentation method for strawberry instances that improves the Mask R-CNN network to reduce computational costs in the inference phase. For this study, a new high-resolution strawberry image dataset was obtained and a new performance metric was proposed: Example intersection ratio (I2oU), the experimental results show that the proposed method significantly reduces the inference time while maintaining the average precision (mAP) and average I2oU indexes comparable to the original Mask R-CNN.

As mentioned earlier, the instance segmentation technique is widely used in the vision system of agricultural robots. However, the color changes of tomato fruits in different ripening stages are subtle, especially under natural lighting conditions, which makes it difficult to distinguish. In addition, the shape, size and growth posture of the fruit are different, which is easy to overlap and occlusion, further increasing the difficulty of detection (Gongal et al., 2015). In the aspect of fruit segmentation, the traditional segmentation method is difficult to be effective because the surface of tomato is smooth and the texture characteristics are not obvious. Moreover, accurate robot picking requires accurate positioning and high-precision contour information, which puts forward higher requirements for the accuracy and efficiency of the instance segmentation algorithm. Therefore, this paper aims to study the method of tomato ripened detection and fruit segmentation based on instance segmentation. In view of the challenges existing in the task, this paper proposes two innovative modules to improve the performance of the model. The main contributions of this paper are as follows:

- ACP-Tomato-Seg model was proposed for tomato ripeness detection and fruit segmentation. The model integrates three modules, AOFRM, CMPRD and PSA, which can effectively solve the challenges brought by the change of tomato shape, size and occlusion, and improve the detection ability and fruit segmentation accuracy of the model for tomatoes with different maturity levels.
- AOFRM module: Used to enhance the model’s perception of tomato targets. The module captures the shape change and orientation features of tomatoes by deformable convolution and asymmetric convolution respectively, thereby enhancing the feature representation and enabling the model to better adapt to the diversity of tomato targets.
- CMPRD module is used to enrich the scale information of features. The multi-scale feature extraction and fusion strategy are carried out by the self-defined pool kernel, which enables the model to focus on the local details and the overall contour of the tomato target at the same time, thus improving the detection accuracy of different sizes of tomatoes.
- A dataset of large and small tomatoes with six ripeness categories was constructed. The data set can be used to train and evaluate tomato ripeness detection and fruit segmentation models, and provide data support for related studies.

## 2 Materials and methods

### 2.1 Production of data sets

#### 2.1.1 Data sample collection

**Figure 1:**
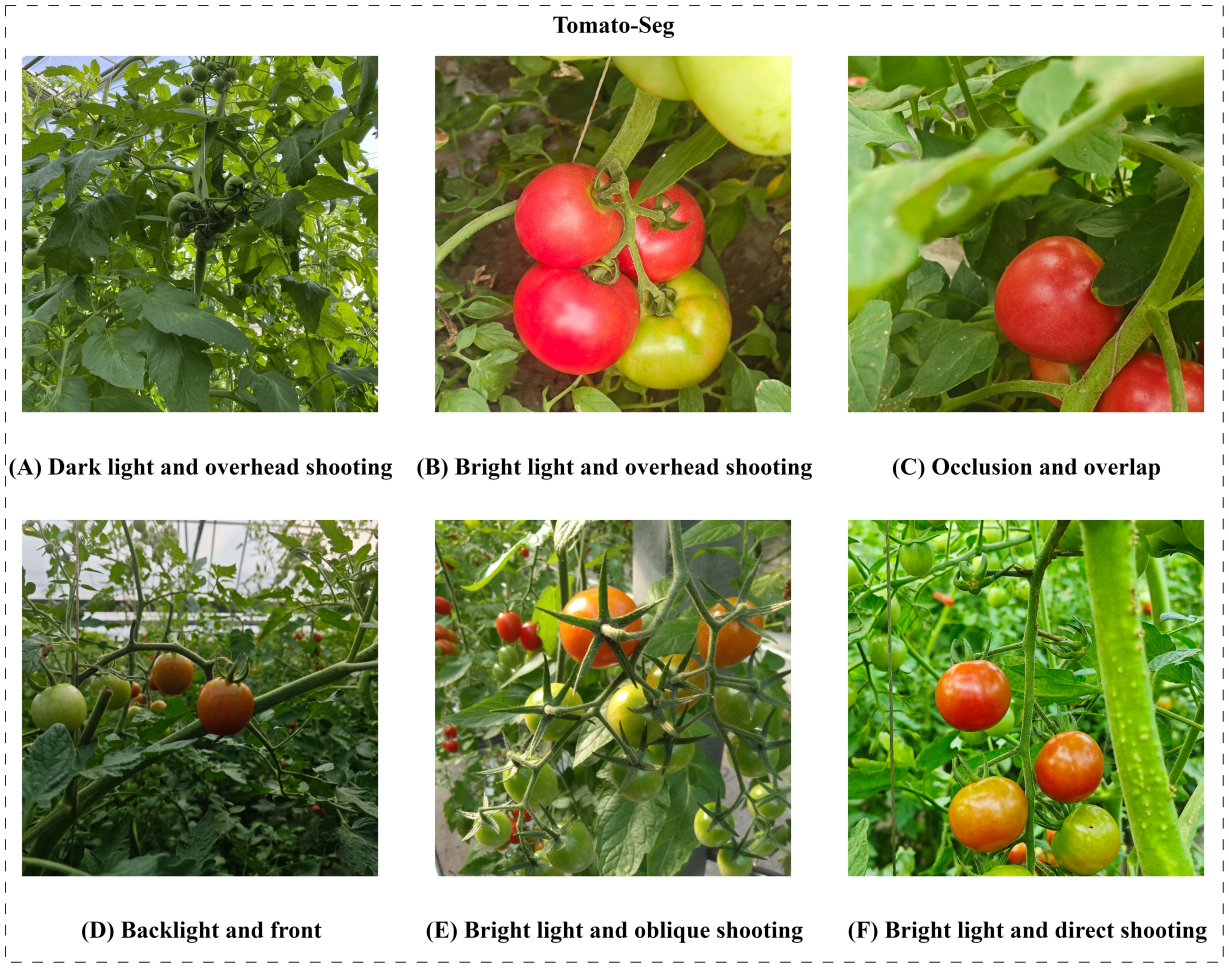
In order to construct a tomato dataset covering different lighting conditions, shooting angles and occlusion conditions, the following scenarios were considered in the data acquisition process: **(A)** the upshot Angle under low light conditions; **(B)** Overhead shooting Angle under sufficient light; **(C)** The shooting Angle under occlusion and overlap; **(D)** Frontal shooting under backlight; **(E)** Oblique shooting Angle under sufficient light; **(F)** Front facing shooting under sufficient lighting.

In order to train and evaluate the proposed ACP-Tomato-Seg instance segmentation algorithm, we establish a Tomato image dataset named Tomato-Seg, which is from the tomato experimental greenhouse of Jilin Agricultural University. After rigorous screening, excessive fuzzy, severely overexposed or irrelevant images are removed. A total of 1061 high-resolution color images were collected, covering large and small tomato fruits at different ripening stages, angles, distances, lighting conditions, overlap and occlusion degrees. Some of the images were shown in **Figure 1**. Finally, we randomly divided the data set into the original training set and the original verification set according to 8:2.

#### 2.1.2 Data set enhancement

**Figure 2:**
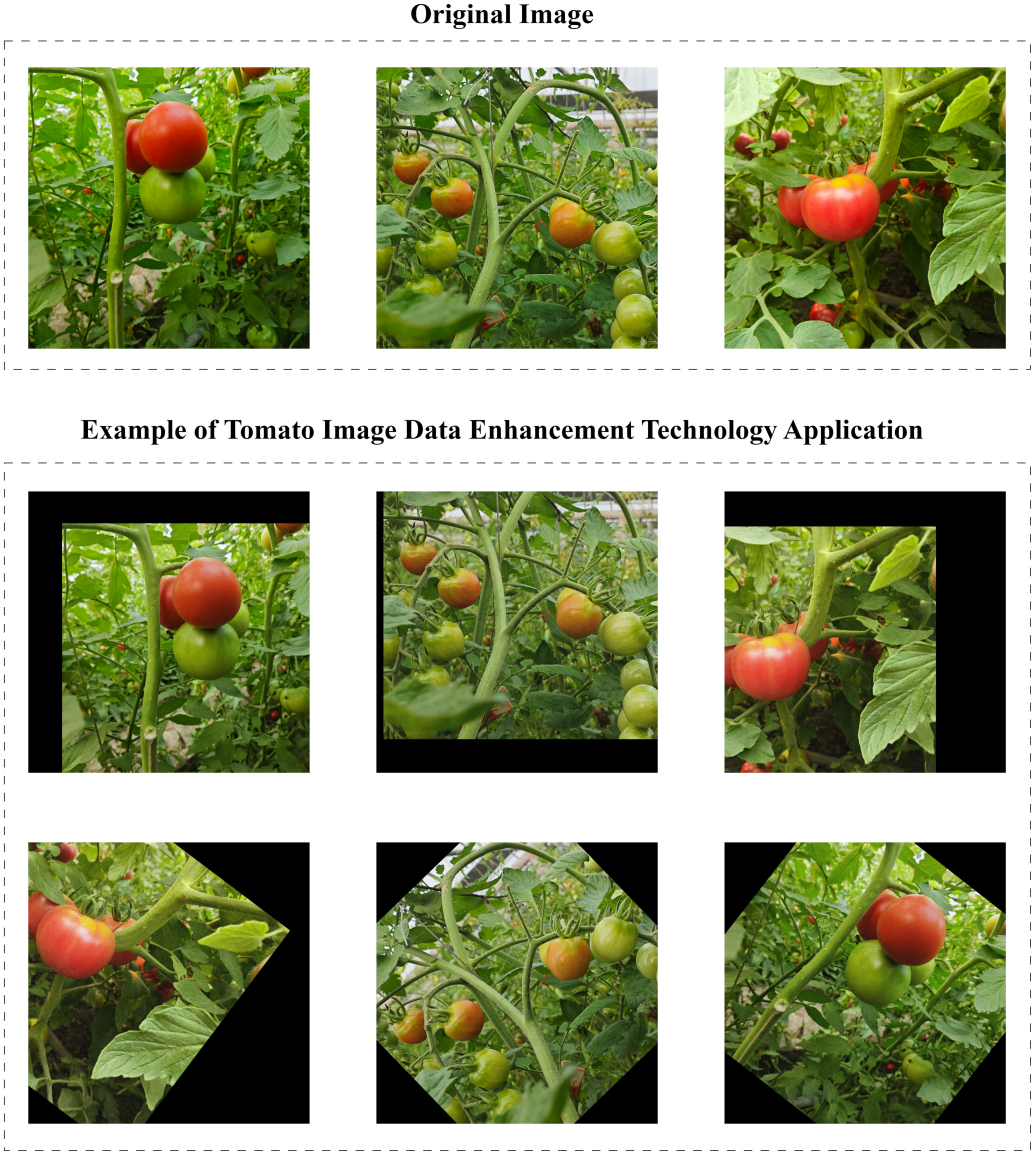
An example of the application of data enhancement techniques, randomly rotating an image around 30 to 45 degrees, cropping, adding noise, and translating.

In order to eliminate potential problems that may occur in the training process of the model, for example, the model overlearns the details and noise in the training data, resulting in poor performance on unseen data and poor generalization ability. The model may also over-rely on certain features in the training data, such as specific lighting conditions, shooting angles, or tomato morphology, and the model’s detection and segmentation performance will deteriorate when confronted with tomato images with different lighting, angles, or morphology. We implement the data enhancement strategy on the training set of Tomato-Seg dataset, and carry out a series of random transformations on the original image to effectively expand the diversity of training samples (Arnal Barbedo, 2019). These transformations include 30 to 45 degrees of random left-to-right rotation, random cropping, adding random noise, and translation to simulate real-world changes in tomato growing posture, lighting conditions, and shooting angles, as shown in **Figure 2**. Finally, through the application of data enhancement techniques, we expanded the training set by 4 times. **Table 1** shows the proportion of each instance in the training set and the test set.

**Table 1:** Shows the composition of the data set, including the number of images of the original training set, the enhanced training set and the verification set, as well as the number of instances of the six categories.

Train			Val
Images Instances	Original (848)	Enhance (2544)	Original (213)
Large-fully-mature	584	1752	128
Large-semi-mature	633	1899	139
Large-immature	1500	4500	354
Small-fully-mature	1025	3075	246
Small-semi-mature	854	2562	262
Small-immature	3592	10776	1006
All	8188	24564	2135

The six categories are: the full ripening stage of large tomatoes, the semi-ripening stage of large tomatoes, the immature stage of large tomatoes, the full ripening stage of small tomatoes, the semi-ripening stage of small tomatoes and the immature stage of small tomatoes.

### 2.2 Model improvement

**Figure 3:**
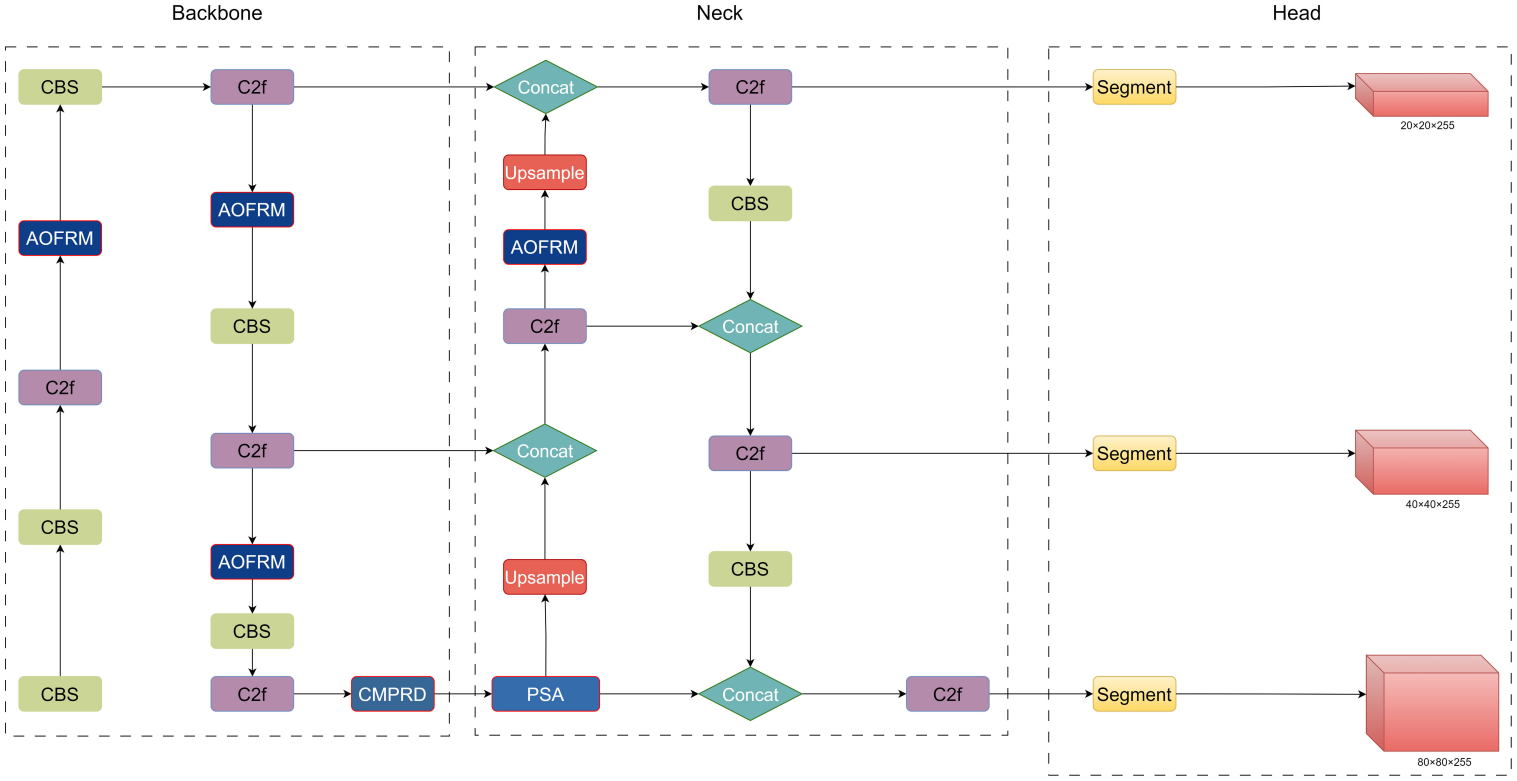
Network architecture of ACP-Tomato-Seg model.

ACP-Tomato-Seg model adopts the advanced YOLOv8-seg (Jocher et al., 2023) model as the basic architecture, which is composed of trunk extraction network, neck and segmentation head respectively. In order to enhance the segmentation ability of the model in the case of overlap and occlusion of tomatoes, we added the adaptive and oriented feature refinement module (AOFRM) to the C2f module of the backbone extraction network to enhance the model’s perception ability of tomato targets and provide more discriminative feature representation for subsequent modules. In order to further improve the detection ability of tomato maturity of different sizes, the original SPPF module was replaced by a custom multi-scale pooling module (CMPRD) with residual and depth. In order to guide the model to pay attention to important feature information and suppress the interference of irrelevant information, so as to improve the detection and segmentation accuracy of the model, the PSA module is introduced between the backbone extraction network and the neck. The improved algorithm model structure is shown in **Figure 3**:

#### 2.2.1 An adaptive and oriented feature refinement module

**Figure 4:**
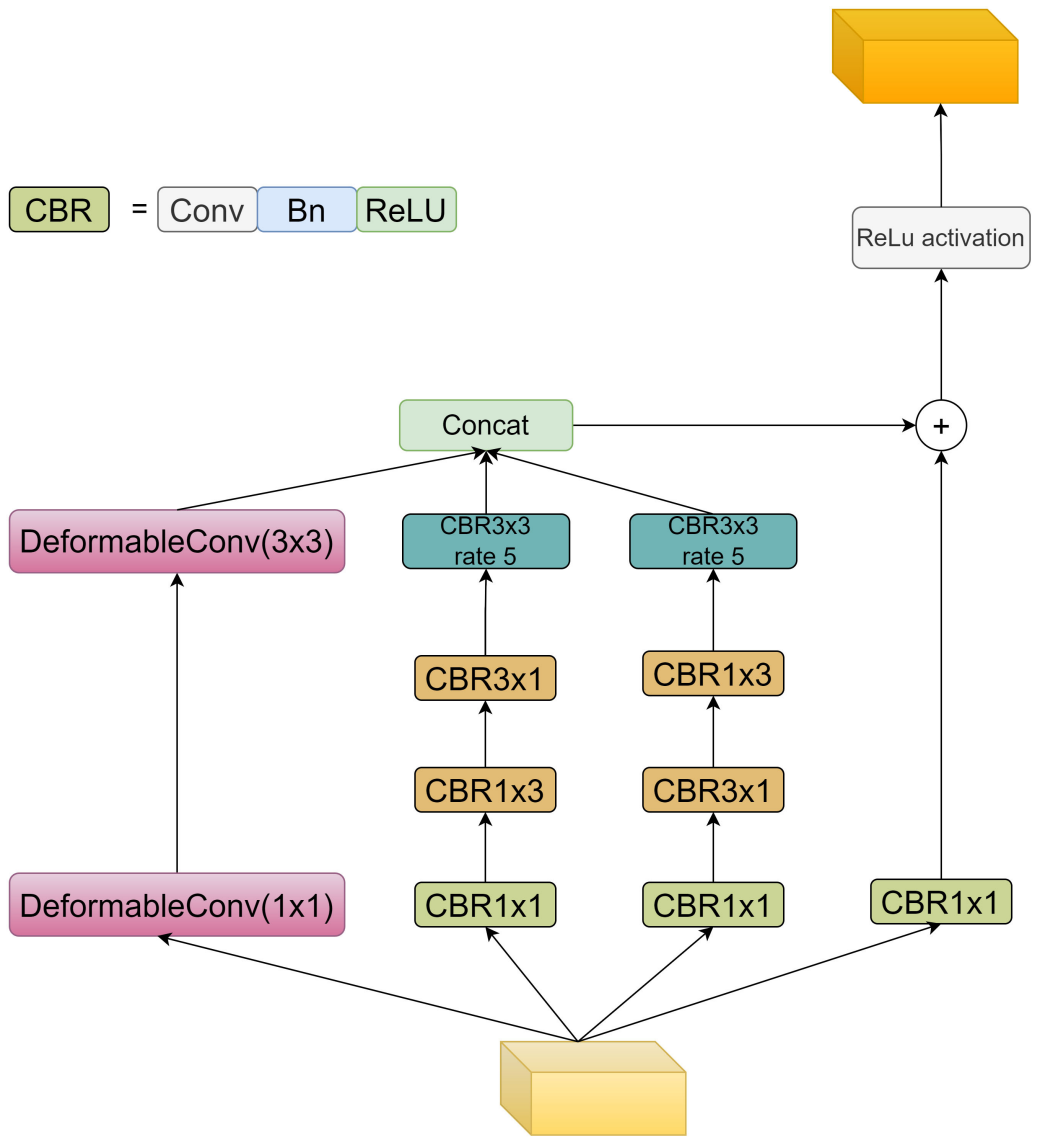
Network structure of AOFRM module.

To enhance feature representation and improve segmentation, an adaptive and oriented feature refinement module (AOFRM) is proposed and integrated into YOLOv8s-seg architecture. Unlike previous feature enhancement methods, the ADFRM module does not simply stack convolutions or attention mechanisms. Instead, it differentially designs adaptive shape modeling and multi-directional feature capture capabilities in response to the unique challenges of the tomato instance segmentation task. This module is strategically placed after the c2f feature extraction module of the backbone network to take full advantage of its rich semantic and spatial information. The design essence of the ADFRM module is based on two core principles: adapting to shape changes and capturing multi-directional features. These two principles are achieved by integrating deformable convolution (Zhu et al., 2018) and strategically designed asymmetric convolution (Ding et al., 2019) in a multi-branch architecture. **Figure 4** shows the detailed structure of the AOFRM module:

AOFRM module adopts three-branch structure to realize self-adaptation and effective fusion of direction information. Among them, adaptive feature extraction (Branch 0) utilizes two cascaded deformable convolution layers. Different from traditional convolution, deformable convolution provides a dynamic receptive field by learning the offset of the sampling grid, and its formula can be expressed as:


(1) $$\begin{array}{c} \text{y}(\text{p})=\sum_{\text{k}=1}^{\text{K}}{\text{W}}_{\text{k}}\cdot x(\text{p}+{\text{p}}_{\text{k}}+\text{Δ}{\text{p}}_{\text{k}}) \end{array}$$

Where, y(p) represents the eigenvalue of the output feature map at position *p*, w(k) represents the weight of the convolutional kernel at position *k*, $x(p+p_{k}+\text{Δ}p_{k})$ represents the eigenvalue of the input feature map at position $\left(p+p_{k}+\text{Δ}p_{k}\right)$ , and $p_{k}$ represents the regular sampling position of the convolutional kernel, $\text{Δ}p_{k}$ represents the learned offset. This adaptability enables branch 0 to effectively capture the shape of the tomato regardless of its orientation, growth stage, or shade degree, thus achieving robust segmentation performance under real conditions. Horizontal feature enhancement (branch 1) and vertical feature enhancement (branch 2) adopt the asymmetric convolution kernel of (1, 3) and (3, 1), and the convolution sequence of the two branches is opposite, so as to effectively extract the directional features of the target. The formula can be expressed as follows:


(2) $$\begin{array}{c} \text{y}(\text{p})=\sum_{\text{i},\text{j}}\text{w}(\text{i},\text{j})\cdot \text{x}(\text{p}+(\text{i},\text{j})) \end{array}$$

Where, y(p) represents the eigenvalue of the output feature map at position *p*, w(i, j) represents the weight of the convolution kernel at position (i, j) , and x(p + (i, j)) represents the eigenvalue of the input feature map at position p + (i, j) . In addition, both branches use 3x3 Dilated convolution (Yu and Koltun, 2015) (dilation=5) to expand the receptive field to better extract information such as the width, height, texture, and edge of the target, a configuration that is crucial for accurately delineating tomato boundaries and contours, especially in the case of partial occlusion. The features extracted from the three branches are connected along the channel dimension, effectively fusing adaptive and directional information. This connected feature map is then passed through a 1x1 convolution layer to reduce dimensionality and refine the representation, resulting in an information-rich feature map tailored for tomato instance segmentation. In order to solve the problem of disappearing gradients and facilitate the seamless flow of information, residual connectivity is added to the AOFRM module. This connection adds input features directly to output features, facilitating gradient propagation and enhancing the learning process during backpropagation.

The ADFRM module effectively captures global shape variations and local directional cues by innovatively combining deformable convolution, asymmetric convolution, and dilated convolution, and adopting a unique three-branch structure and feature fusion strategy, thereby significantly enhancing the feature representation for tomato instance segmentation. This enhanced feature representation will help generate more accurate and smoother tomato contours, minimize instances of incorrect segmentation, and ultimately improve the overall segmentation quality.

#### 2.2.2 Custom multi-scale pooling with residuals and depth

**Figure 5:**
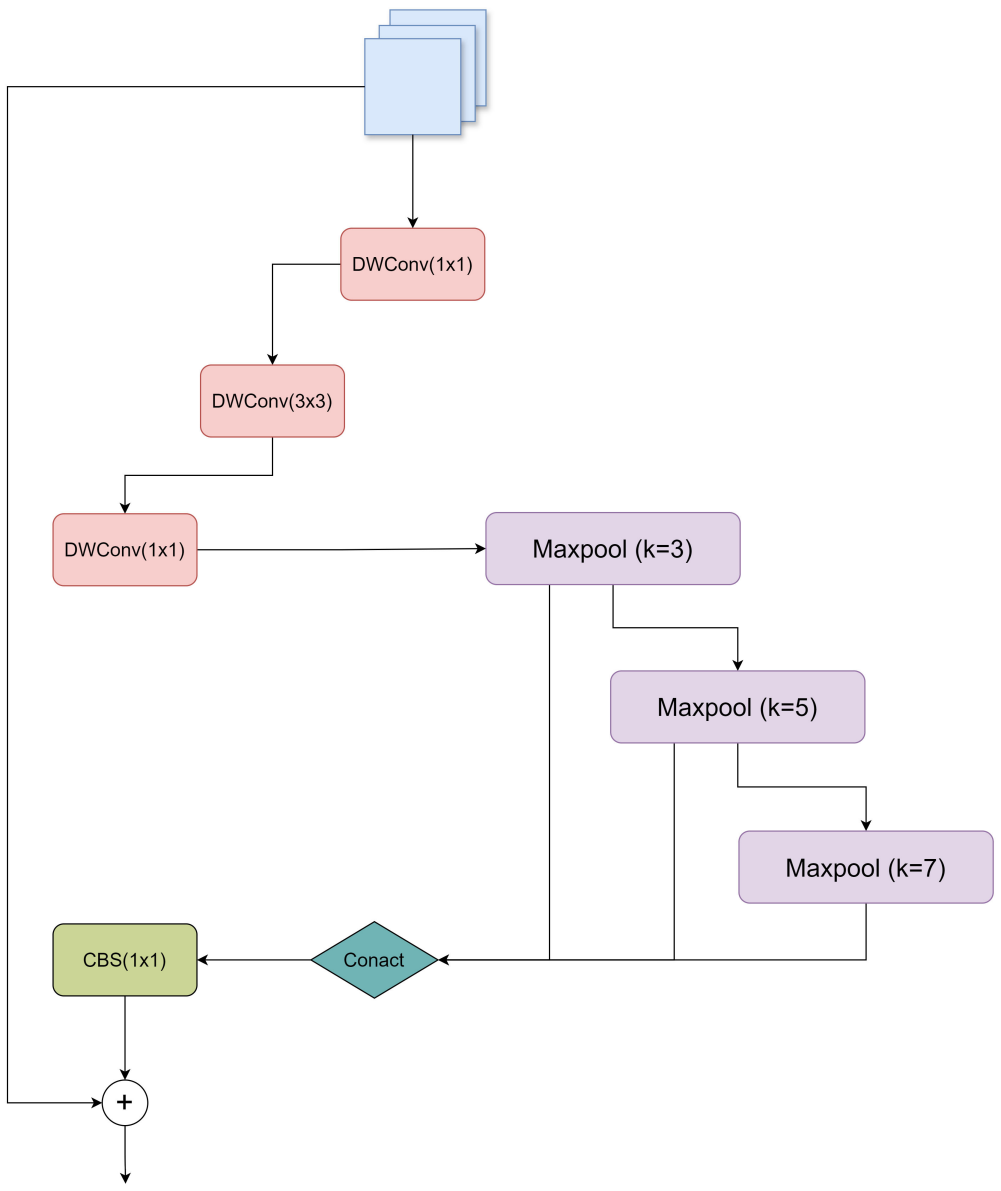
Network structure diagram of CMPRD module.

Multi-scale feature extraction and fusion are crucial for various computer vision tasks (Gao et al., 2021). In order to capture features at different scales, SPP (He et al., 2014) has been proposed and widely used in various CNN architectures. SPP uses multiple parallel pooling layers, each with a different fixed size, to extract multi-scale features. However, the computational cost of SPP is high, and the efficiency of information fusion between different pooling layers is limited. To solve these problems, SPPF (Jocher, 2020) is proposed. SPPF achieves the same functionality as SPP in a cascading manner, thereby increasing efficiency. However, SPPF still relies on simple concatenation operations to fuse features extracted from different pooling layers, which may not be optimal. In order to further improve the model’s ability to detect the maturity of tomatoes of different sizes, a custom multi-scale pooling module with residual and depth, called CMPRD, was proposed in this paper, as shown in **Figure 5**:

CMPRD module consists of main branch, custom multi-scale pooling branch, feature fusion and residual connection:

The main branch is constructed with three successive deep separable convolution layers (DWConv) (Howard et al., 2017), whose formula can be expressed as:


(3) $$\begin{array}{c} \text{y}(\text{p})=\sum_{\text{k}}{\text{w}}_{\text{d}}(\text{k})\cdot \text{x}(\text{p}+{\text{p}}_{\text{k}}) \end{array}$$

(4) $$\begin{array}{c} \text{y}(\text{p})=\sum_{\text{c}}{\text{w}}_{\text{p}}(\text{c})\cdot {\text{y}}_{\text{d},\text{c}}(\text{p}) \end{array}$$

Where, y(p) represents the eigenvalue of the output feature map at position *p*, $w_{d}(k)$ represents the weight of the deep convolutional kernel at position *k*, $x(p\;+\;p_{k})$ represents the eigenvalue of the input feature map at position $p\;+\;p_{k}$ , and $w_{p}(c)$ represents the weight of the point-by-point convolutional kernel at channel *c*. $y_{c}(p)$ represents the eigenvalue of the deep convolutional output feature map at channel c and position *p*. First, a 1x1 DWConv layer is used to reduce the number of channels in the input feature map, reducing the amount of computation. A 3x3 DWConv layer then performs more refined feature extraction. Finally, another 1x1 DWConv layer restores the channel count to the input dimension. The use of depth-separable convolution effectively reduces the number of parameters and computation, making the model more lightweight and efficient.

Custom multi-scale pooling branches: Unlike the fixed pooling kernel size of SPP and SPPF, multiple pooling operations are performed, and the diversity of the receptive field is relatively limited. The adaptive multi-scale pooling branch of the CMPRD module can customize the kernel size (3x3, 5x5, 7x7), which allows us to flexibly adjust the size of the receptive field of each pooling layer according to the task requirements, extract the feature information of different scales, obtain a richer combination of receptive fields, and better capture the target features of different scales in the image. Therefore, it can better adapt to different target scales and scene complexity.

Feature fusion and residual joining: In order to effectively fuse the features extracted from the main branch and multi-scale pooling branch, the CMPRD splices their outputs and uses a 1x1 convolution layer for reduction and feature fusion. In addition, in order to promote network training and information flow, the CMPRD module also introduces residual connection, adding the elements at the corresponding positions of the input features and the fused features to further enhance the learning ability of the model.

The CMPRD module overcomes the limitations of traditional SPP/SPPF modules in flexibility, efficiency, and fusion methods by innovatively introducing a custom multi-scale pooling branch, an efficient depthwise separable convolution main branch, and a refined feature fusion and residual connection mechanism. It extracts and fuses multi-scale features more effectively, thereby significantly improving the performance of the target detection model in complex scenes and for multi-scale targets.

#### 2.2.3 PSA

**Figure 6:**
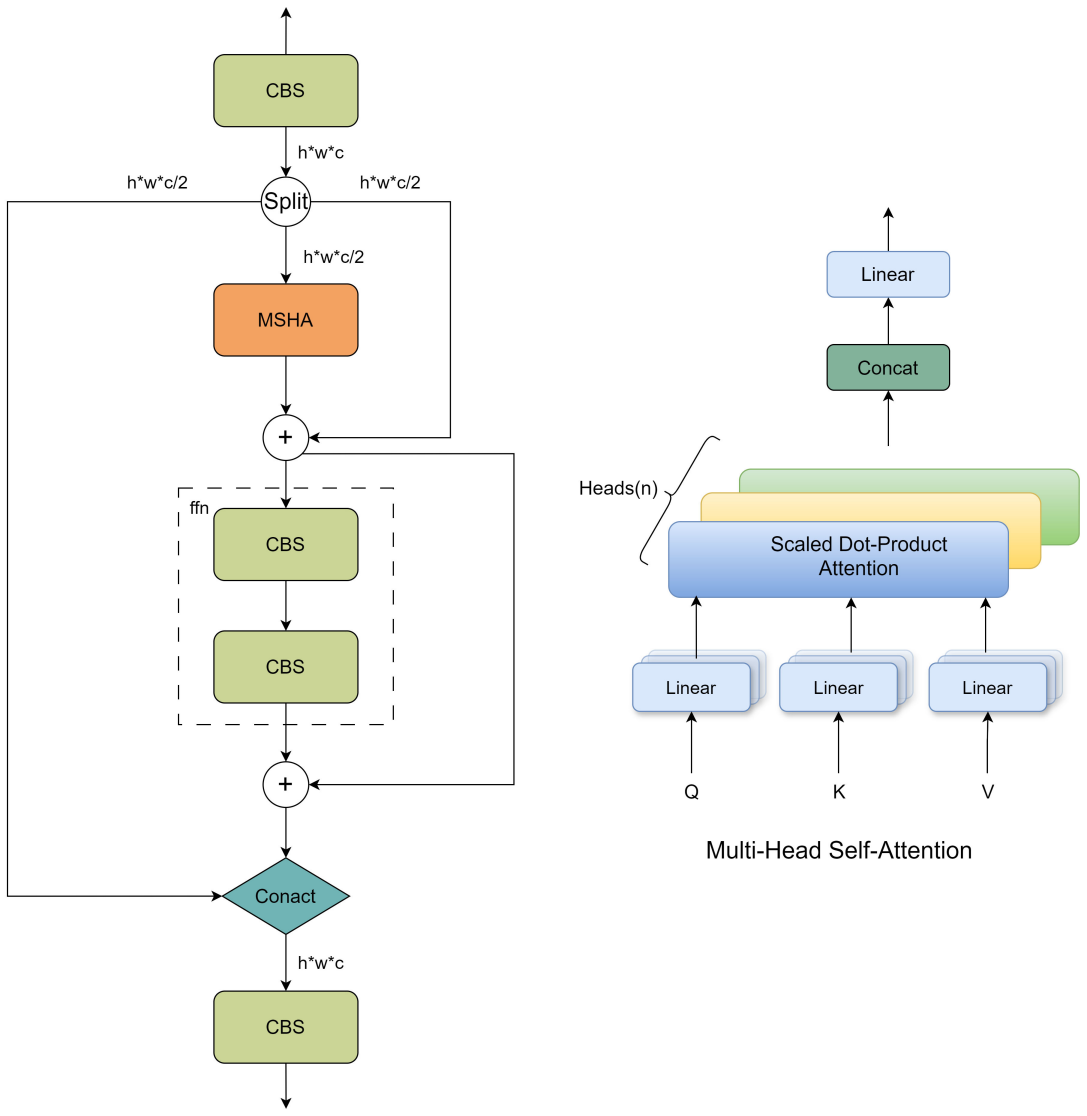
Network structure diagram of PSA module.

Although the CMPRD module proposed in this paper can effectively increase the receptive field through the custom multi-scale pooling branch, its main concern is the fusion of local features, and it lacks the capture of global context information. In order to pay attention to both local features and global context information, and avoid the high computational cost brought by traditional self-attention mechanism, this paper introduces a Partial self-attention module (PSA) based on YOLOv10 (Wang A. et al., 2024). It is embedded between the backbone network and the neck network of ACP-TomatoSeg network to enhance the ability of the model to extract tomato features. The module structure is shown in **Figure 6**. The core idea of PSA module is to divide the input feature map into two parts for local feature extraction and global context information modeling respectively.

PSA module combines channel attention and spatial attention mechanism, and can focus on the spatial dimension and channel dimension information of feature map at the same time. Among them, channel attention is used to screen the feature channels that are more important for the detection of tomato maturity, while spatial attention is used to focus on the tomato-related regions in the image, thereby improving the sensitivity of the model to the target information. The MHSA (Vaswani et al., 2017) module is nested inside the PSA module to capture the relationship between different positions in the feature map and further enhance the model’s ability to extract details such as tomato contour and texture. Compared with the commonly used channel-spatial attention modules CBAM (Woo et al., 2018) and Coordinate Attention (Hou et al., 2021), the core innovation of the PSA module lies in the idea of “partial self-attention” and the combination of global context modeling and local feature preservation. Different from attention mechanisms such as CBAM and CA that mainly focus on the selective enhancement of the channel or spatial dimension, the PSA module focuses more on explicitly modeling the global context information of the feature map through the MHSA module and fusing it with local features. At the same time, by only performing global context modeling on a portion of the feature maps, the PSA module effectively reduces the computational complexity, achieving a better balance between computational efficiency and performance improvement. Furthermore, the design concept of the PSA module is more in line with the human visual attention mechanism, that is; while paying attention to local details, it is also necessary to understand the global scene information to make accurate judgments.

Specifically, the PSA module first uses a 1x1 convolution to reduce the number of channels of the input feature map, achieving the screening of channel information by controlling the number of channels, and then divides it into two parts, A and B. Among them, part A is directly sent to the subsequent module to preserve local feature information and effectively reduce the amount of calculation, while part B of the feature map is sent to the nested PSA (Partial Self-Attention) block for global context information modeling. The core component of the PSA block is the Multi-Head Self-Attention (MHSA) module, which calculates the correlation between any two points in the feature map to obtain an attention matrix used to represent the importance between different positions. The formula is as follows:


(5) $$\begin{array}{c} {\text{B}}^{\prime }=\text{B}+\text{softmax}(\frac{QK^{T}}{\sqrt{d_{k}}})\times V \end{array}$$

Where Q, K, V are the query, key, and value matrices, respectively, obtained by linear transformation, and *d_k_* is the dimension of the key. The PSA module, unlike traditional spatial attention mechanisms, can more effectively pay attention to the spatial and channel dimension information of the feature map simultaneously. Additionally, the PSA module also contains a feed-forward network (FFN):


(6) $$\begin{array}{c} B^{\prime \prime }=B^{\prime }+\text{FFN}(B^{\prime }) \end{array}$$

Finally, the two feature maps A and B'' are concatenated, and the number of channels is restored through a 1x1 convolution to obtain the final output feature map Y:


(7) $$\begin{array}{c} Y=\text{Con}v_{1\times 1}(\text{concat}(A,B^{\prime \prime })) \end{array}$$

By introducing the attentional mechanism module, ACP-Tomato-Seg can locate the tomato target more accurately and extract more fine feature information, thus improving the accuracy of tomato ripenness detection and fruit segmentation.

### 2.3 Evaluation indicators

The basic evaluation indexes used in this study include accuracy (P), recall rate (R) and average accuracy (mAP50 and MAP50-95). The model’s performance in tomato ripen detection and fruit segmentation tasks was evaluated from two aspects: Box and Mask. For the riper detection task, P and R indexes were used to evaluate the model’s positioning accuracy and recall rate of tomato target borders with different maturity levels, while mAP50 and MAP50-95 indexes were used to evaluate the model’s average accuracy under different IoU thresholds. For the fruit segmentation task, we adopted the same index system, represented by the indexes Mask P, Mask R, Mask mAP50 and Mask mAP50-95, respectively, to evaluate the model’s performance of tomato segmentation at the pixel level. The calculation formulas for each evaluation indicator are as follows:


(8) $$\begin{array}{c} P_{Box/Mask}=\frac{TP}{TP+FP} \end{array}$$

(9) $$\begin{array}{c} R_{Box/Mask}=\frac{TP}{TP+FN} \end{array}$$

(10) $$\begin{array}{c} AP_{Box/Mask}=\int_{0}^{1}p(r)dr \end{array}$$

(11) $$\begin{array}{c} mAP_{Box/Mask}=\frac{\sum_{I=1}^{N}AP_{i}}{N} \end{array}$$

where Box represents the target box, Mask represents the mask, TP represents the case in which the model correctly classifies the actually positive samples as positive, FP represents the case in which the model incorrectly classifies the actually negative samples as positive, and FN refers to the case in which the model incorrectly classifies the actually positive samples as negative.

## 3 Results and analysis

### 3.1 Experimental environment and parameter setting

This study uses the same equipment for experiments, and the model is based on PyTorch deep learning framework and developed in Anaconda environment. **Table 2** shows the main experimental equipment environment configuration. The experimental hyperparameters are set as follows: the number of iterations is 200, the batch size is 64, the optimizer is SGD, the initial learning rate is 0.01, the learning rate momentum is 0.937, the weight attenuation coefficient is 0.0005, and the model is trained using two Gpus.

**Table 2:** Experimental environment configuration.

Environment Configuration	Parameter
Operating System	Linux
CPU	Intel(R) Xeon(R) Gold 6148 CPU @ 2.40 GHz
GPU	2×A100(80 GB)
Development environment	PyCharm 2023.2.5
Language	Python 3.8.10
Framework	PyTorch 2.0.1
Operating platform	CUDA 11.8

### 3.2 Experimental results of ACP-tomato-seg model

**Figure 7:**
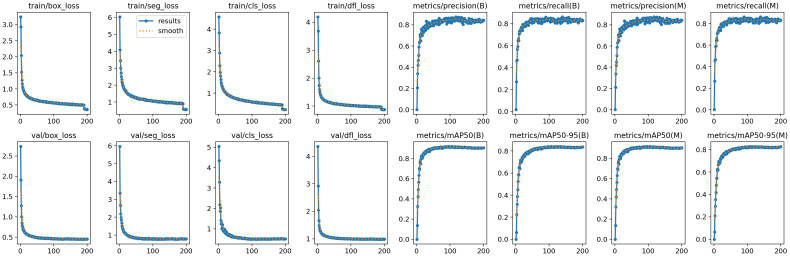
Experimental results of ACP-Tomato-Seg model.

**Figure 7** intuitively shows the experimental results of the ACP-Tomato-Seg model on the Tomato-Seg data set, which completely records the results of the ACP-Tomato-Seg model during the training process. The variation of the different loss indicators and the Precision, Recall and average accuracy under the two categories of bounding box and mask.

**Figure 8:**
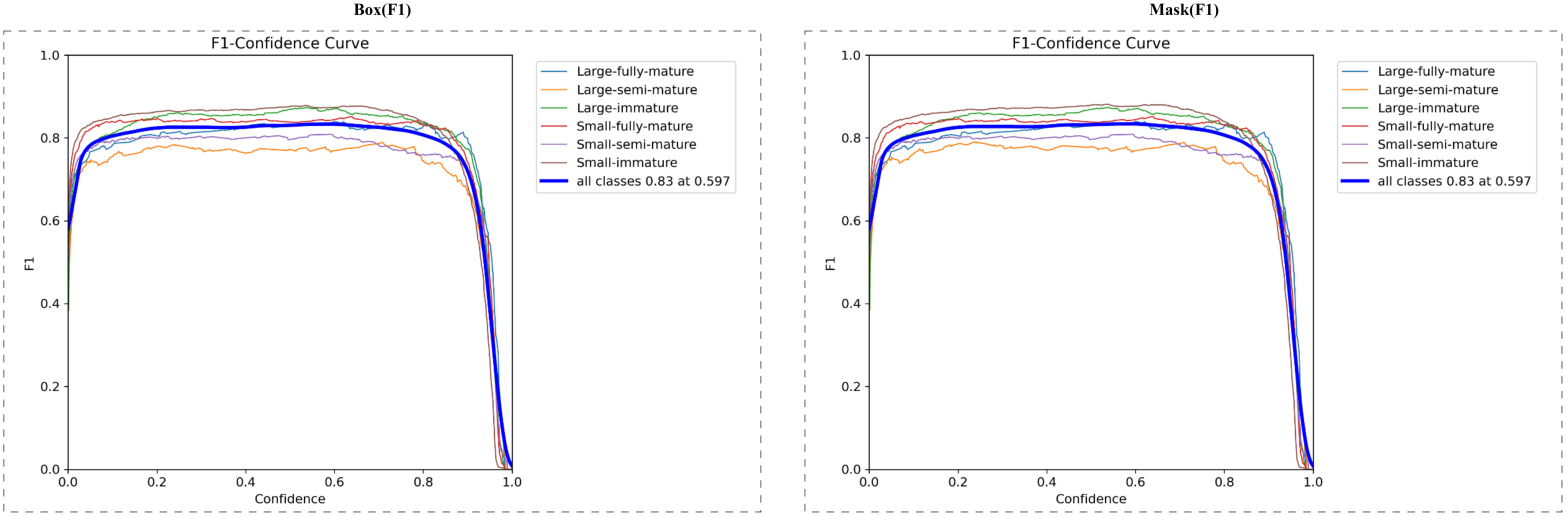
F1 scores under box and mask.

**Figure 8** shows the F1 score curve of each tomato maturity category under different confidence thresholds. F1 score is an important indicator to measure the comprehensive performance of the model, which comprehensively considers the accuracy and recall rate of the model.

**Figure 9:**
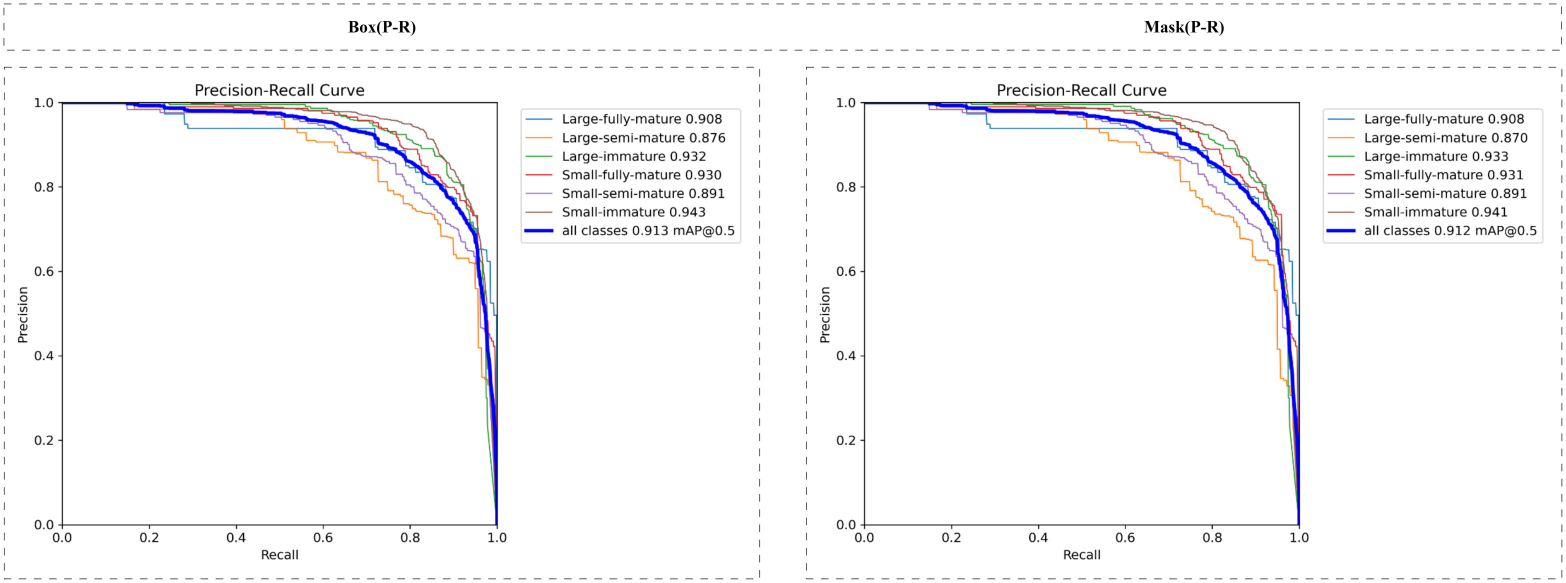
P-R curve under box and mask.

**Figure 9** shows the P-R curve of the model, which intuitively reflects the tradeoff between the accuracy rate and the recall rate of the model under different confidence thresholds. Ideally, we want the model to achieve both a high accuracy rate and a high recall rate, i.e. the closer the P-R curve is to the top right corner, the better. As can be seen from the figure, the P-R curve of the model is smooth on the whole, and still maintains a high accuracy rate under a high recall rate, which indicates that the model can effectively control the false detection rate while ensuring the recall rate, and achieve a good recognition effect.

### 3.3 Comparative experiments of different models

To evaluate the model performance, we selected multiple mainstream models for comparative experiments to verify ACP-Tomato- Seg advantages in the riper detection and fruit segmentation tasks of large and small tomatoes. These include the Mask R-CNN (He et al., 2017), Mask2Former (Cheng et al., 2021),RT-Detr series (Lv et al., 2023), the YOLO series [v3-tiny (Redmon and Farhadi, 2018), v5m-seg (Jocher, 2020), v6-seg (Li et al., 2022), v7 (Wang C-Y. et al., 2022), v8s-seg (Jocher et al., 2023), v9c-seg, v9e-seg (Wang C-Y. et al., 2024), v10s-seg (Wang A. et al., 2024), 11s-seg (Jocher et al., 2023), v12s-seg (Tian et al., 2025)], and the ACP-Tomato- seg (our). **Table 3** shows the evaluation of each model on the two tasks of Box and Mask:

**Table 3:** 12 Performance comparison of mainstream models.

Model	Box				Mask			
	P	R	mAP50	mAP50-95	P	R	mAP50	mAP50-95
Mask R-CNN	80.2	79.8	85.4	74.1	80.5	80.3	86.0	74.3
Mask2Former	83.6	80.2	89.1	75.8	83.2	81.1	89.0	76.9
RT-Detr-l	83.4	81.4	88.8	78.2	83.4	81.4	88.7	74.6
RT-Detr-resnet50	83.0	81.4	89.4	78.7	83.0	81.3	89.2	74.7
YOLOv3-tiny	78.2	71.8	77.0	60.0				
YOLOv5m-seg	80.6	81.6	85.5	77.0	80.6	81.4	85.3	73.2
YOLOv6-seg	81.3	79.1	86.5	74.6	81.2	79.0	86.2	71.4
YOLOv7	82.8	81.1	87.1	71.5				
YOLOv8s-seg	84.2	75.8	85.7	76.2	81.5	78.4	85.4	74.8
YOLOv9c-seg	83.0	83.2	89.1	81.3	83.0	82.5	89.0	77.7
YOLOv9e-seg	82.6	83.3	89.6	79.6	82.7	83.4	89.6	75.8
YOLOv10s-seg	85.6	80.4	89.4	79.6	86.0	80.1	89.0	76.2
YOLO11s-seg	84.6	80.6	90.5	79.9	84.1	80.4	89.6	81.5
YOLO12s-seg	85.1	81.4	89.9	81.2	85.3	81.8	88.9	80.9
our	85.8	82.2	91.3	84.5	85.5	82.1	91.2	83.3

**Figure 10:**
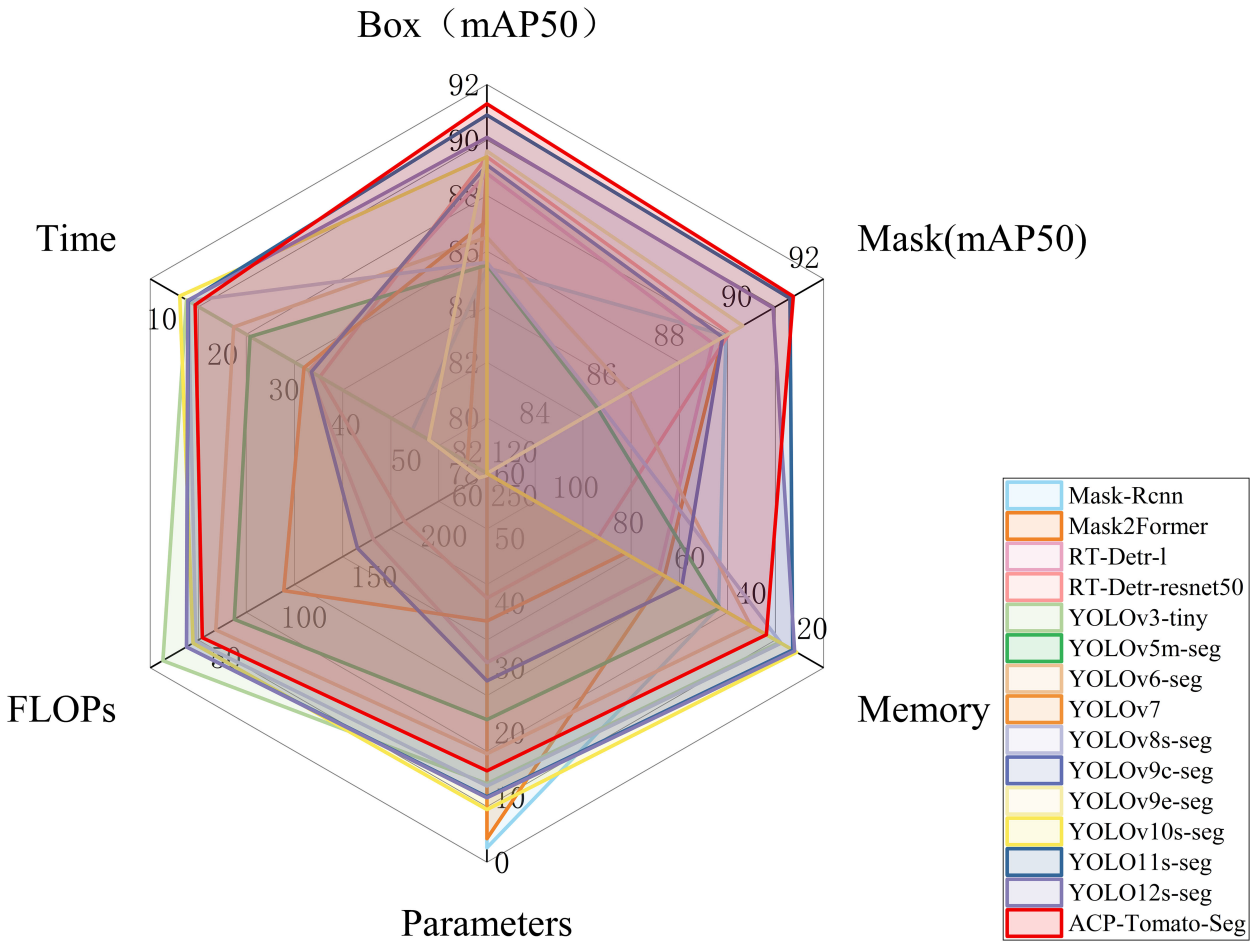
Shows the comprehensive performance comparison of 15 models on multiple indicators, including mAP50 under Box and Mask categories, model size, parameter number, computation amount and average inference time. Each curve in the radar map represents a model, and the closer the intersection of the curve and each axis is to the outside, the better the index. The larger the area enclosed by the curve, the better the comprehensive performance of the model.

**Figure 10** shows the comprehensive performance of different models.

It can be seen that the mAP index of the ACP-Tomato-Seg model proposed in this paper is superior to other comparison models under the two categories of Box and Mask. In addition, it maintains a good balance in terms of memory usage, parameter number and computation amount. Specifically, under Box task, the improved model mAP50 and MAP50-95 reached 91.3% and 84.5% respectively, which increased by 5.6% and 8.3% compared with the original YOLOv8s-seg model. Under the Mask task, the improved model mAP50 and MAP50-95 reached 91.2% and 83.3% respectively, which increased by 5.8% and 8.5% compared with the original YOLOv8s-seg model. At the same time, the improved model occupies only 28.7 MB, the number of parameters is more than 14 million, the calculation amount is 47.1 GFLOPs, and the average inference time is 12.3 ms, which is slightly increased compared with YOLOv8s-seg, but it is within the acceptable range. As shown in **Table 4**, the RT-Detr series and YOLOv9 series models also perform well on mAP50, but their memory consumption, parameter number, inference speed and computation amount are much higher than the models proposed in this paper. In contrast, the models proposed in this paper strike a better balance between performance, efficiency and model size, and are more suitable for practical application scenarios.

**Table 4:** Comprehensive performance parameters of RT-Detr series and YOLOv9 series models.

Model	Box (mAP50)	Mask(mAP50)	Memory(M)	Parameters	FLOPs(G)	Time(ms)
RT-Detr-l	88.8	88.7	63.8	3.0803202	168.9	30.9
RT-Detr-resnet50	89.4	89.2	83.7	4.0754146	191.1	32.7
YOLOv9c-seg	89.1	89.0	56.3	2.7987104	157.7	31.3
YOLOv9e-seg	89.6	89.6	121.9	5.9686306	244.5	50.5
our	91.3	91.2	28.7	1.4121826	47.1	12.3

### 3.4 Visual comparison of test results

**Figure 11:**
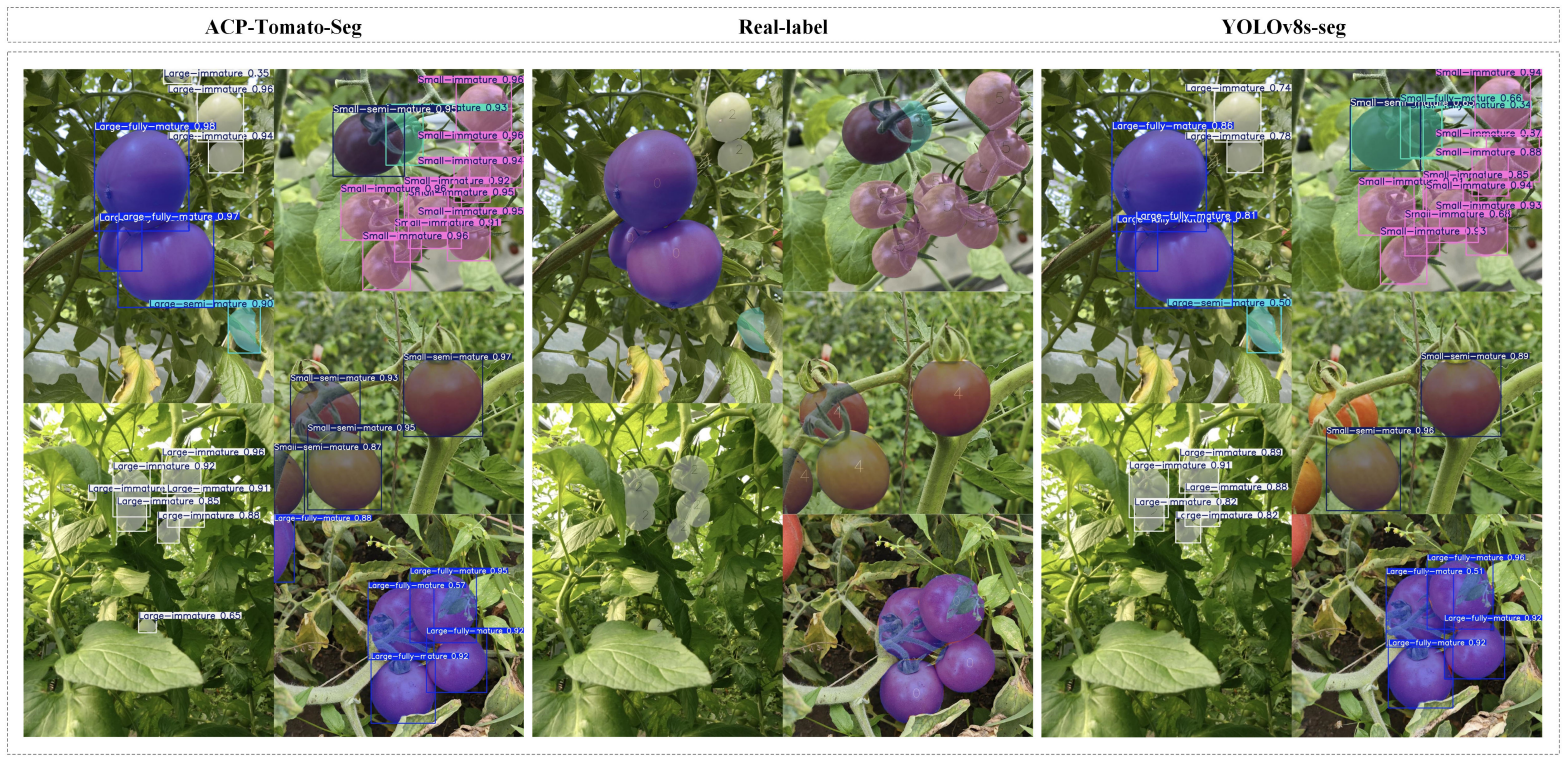
The left side is the detection result of the ACP-Tomato-Seg model, the middle is the real image label, and the right side is the detection result of the original model. It can be seen that compared with the original model, the ACP-Tomato-Seg model can detect and segment the tomato target more accurately.

In order to show the superiority of ACP-Tomato-Seg model more intuitively, we visually compare the detection results of the improved model with the original model. **Figure 11** shows the detection results of some tomato images. As can be seen from the figure, the original model failed to detect partially occluded or overlapping tomatoes. In addition, the original model also had certain shortcomings in segmentation, as the extracted contour was not fine enough and there was deviation from the real contour. In contrast, ACP-Tomato-Seg model can detect and segment tomato targets more accurately. The improved model can effectively identify the occlusion or overlapping tomatoes, and can extract the contour information of tomatoes more accurately, which is more consistent with the real contour. This shows that the improved method proposed in this paper effectively improves the detection accuracy and contour extraction ability of the model, and enhances the robustness of the model in complex scenarios.

### 3.5 Ablation experiment

Experiments in sections 3.2, 3.3 and 3.4 demonstrate the performance superiority of the ACP-Tomato-Seg model. In order to evaluate the effectiveness of the proposed module, we conduct a comprehensive ablation experiment using YOLOv8s-seg architecture as the benchmark model. The ablation experiment was conducted to study the effect of innovative modules on the instance segmentation performance of tomato. **Table 5** shows the comparison between the model after adding the improved module and the original model:

**Table 5:** Results of ablation experiments.

Method	Box				Mask			
	P	R	mAP50	mAP50-95	P	R	mAP50	mAP50-95
base	84.2	75.8	85.7	76.2	81.5	78.4	85.4	74.8
+ADFRM	84.0	80.8	89.3	81.8	84.6	80.1	89.4	80.1
+CMPRD	84.9	81.6	88.2	79.4	84.3	81.8	88.3	78.9
+PSA	84.4	81.2	87.5	78.9	84.1	81.3	87.4	79.0
our	85.8	82.2	91.3	84.5	85.5	82.1	91.2	83.3

#### 3.5.1 Analysis of the influence of AOFRM on model performance

**Figure 12:**
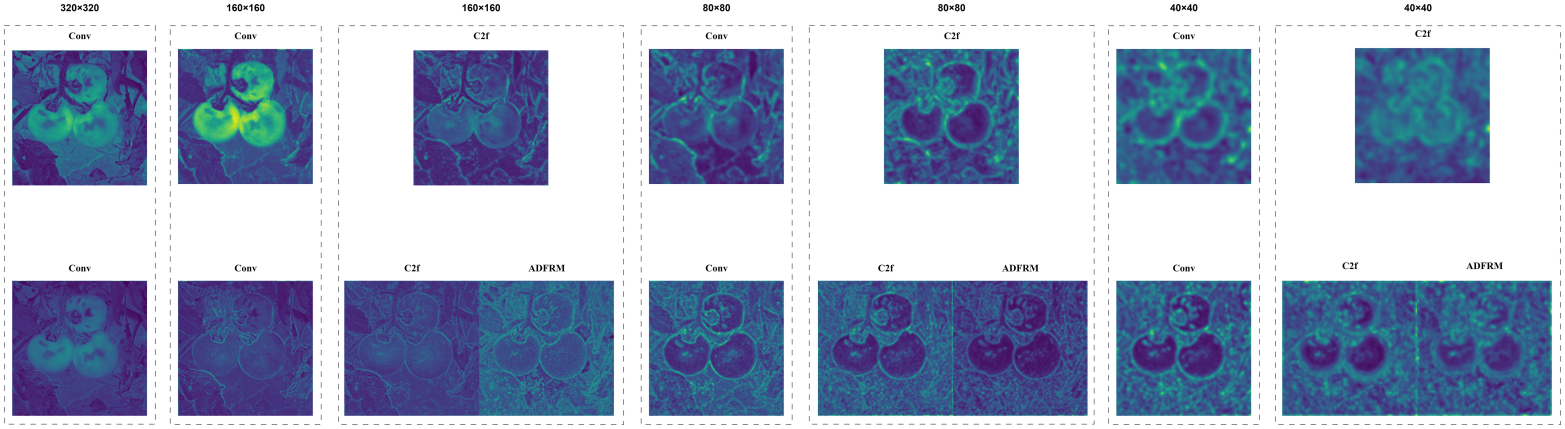
Shows the visual comparison of feature maps between the original model and ACP-TomatoSeg model in different feature extraction stages. Compared with the original model, adding AOFRM after C2f highlights the tomato target more clearly and retains more detailed information, which can better capture the key features of the tomato target and provide more abundant information for subsequent detection and segmentation.

**Table 5** shows the quantitative results of the ablation experiment. Compared with the benchmark model, the mAP50 and MAP50-95 of the model under Box increased by 3.6% and 5.6% respectively, and the mAP50 and MAP50-95 under Mask increased by 4% and 5.3% respectively. To gain a deeper understanding of the impact of the AOFRM module, we visualized the feature maps of each layer downsampled to 40×40 by the baseline model and the improved model. **Figure 12** shows the feature visualization results.

Feature visualization shows that the AOFRM module significantly enhances the clarity and richness of feature representation. Compared to the benchmark model, the AOFRM model presents clearer contours, finer textures, and greater emphasis on tomato-specific features, revealing that the AOFRM module enriches feature representation by capturing the necessary shape and orientation information. The adaptive properties of deformable convolution in the AOFRM module enable the model to deal effectively with the changes of tomato shape, orientation and occlusion. The strategic integration of asymmetric convolution enables the model to capture directional features that are critical to accurately delineating tomato boundaries. The residual connection in the AOFRM module facilitates the seamless flow of information and mitigates the problem of disappearing gradients, helping to enhance learning and improve segmentation performance. It is proved that the AOFRM module proposed can significantly enhance the feature representation of tomato case segmentation.

#### 3.5.2 Impact analysis of CMPRD on model performance

**Figure 13:**
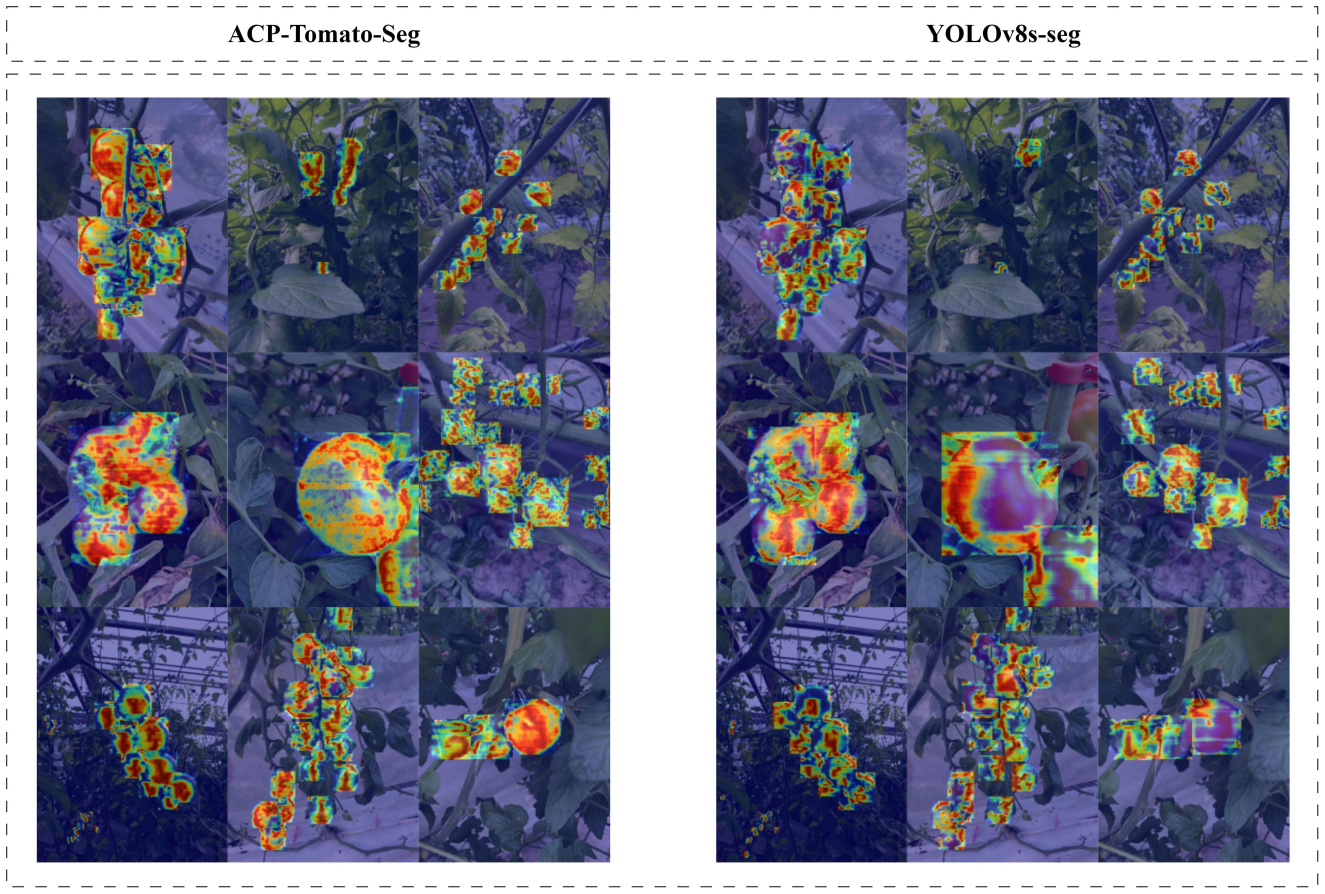
**S**hows the comparison of heat maps in feature extraction between the model with CMPRD module and the original model. Each pixel value on the thermal map represents the activation value of the position, and the higher the activation value, the more likely the target is to appear in the position, which is brighter and more prominent in the thermal map. It can be seen that the heat map generated by the feature map of the CMPRD module is obviously more concentrated on the tomato region than the heat map generated by the original model, indicating that the CMPRD module can effectively enhance the model’s ability to extract tomato features and make it more focused on the target region, thus improving the accuracy of detection and segmentation.

As can be seen from **Table 5**, after the CMPRD module is added, the mAP50 and MAP50-95 of the model under Box are increased by 2.5% and 3.2% respectively, and the mAP50 and MAP50-95 under Mask are increased by 2.9% and 4.1% respectively. In order to further analyze the action mechanism of the CMPRD module, we generated the heatmap of the model on the CMPRD feature layer, as shown in **Figure 13**.

With the addition of the CMPRD module, the model is more focused on the area of concern for the tomatoes and is better able to capture the edges and details of the tomatoes, especially for tomatoes of different sizes and rims. This is mainly attributed to the CMPRD module being able to capture feature information of different scales more flexibly and enhance the model’s ability to extract tomato features, which proves that CMPRD module can effectively enhance the model’s ability to recognize the maturity of tomatoes of different sizes and improve the model’s detection and segmentation performance.

#### 3.5.3 Analysis of the impact of PSA on model performance

In order to verify the effectiveness of PSA module, we add PSA module and several other common attention mechanisms, including SE (Hu et al., 2017), CA (Hou et al., 2021), CBAM (Woo et al., 2018) and ECA (Wang et al., 2019), on the basis of YOLOv8s-seg model respectively, and conduct training and testing on Tomato-Seg dataset. As can be seen from **Table 6**, after the addition of PSA module, the mAP50 and MAP50-95 of the model under Box increased by 1.8% and 2.7% respectively, and the mAP50 and MAP50-95 under Mask increased by 2% and 4.2% respectively. Compared with other attention mechanisms, PSA module also achieved the best performance improvement, which indicates that PSA module can enhance the model’s ability to extract tomato features more effectively than other attention mechanisms, and improve the detection and segmentation performance of the model. The advantage of the PSA module is its ability to combine channel attention and spatial attention mechanisms to extract tomato characteristics more fully. The MHSA module introduced by the PSA module can capture the relationship between different positions in the feature map, and further enhance the model’s ability to extract details such as tomato contour and texture. In addition, the PSA module uses a partial self-attention mechanism to efficiently capture global context information without introducing excessive computational costs. In summary, PSA module can effectively enhance the model’s ability to extract tomato features, improve the model’s detection and segmentation performance, and show its superiority in comparison with other attention mechanisms.

**Table 6:** Comparative experiments of different attention mechanism modules.

Method	Box				Mask			
	P	R	mAP50	mAP50-95	P	R	mAP50	mAP50-95
base	84.2	75.8	85.7	76.2	81.5	78.4	85.4	74.8
+SE	83.2	81.5	86.0	74.3	83.0	81.3	86.1	73.9
+CA	84.6	78.4	86.5	77.1	84.3	78.3	86.2	76.9
+CBAM	84.1	80.0	87.1	77.6	83.9	79.9	86.6	76.3
+ECA	83.6	79.8	86.6	78.0	83.4	78.9	86.3	77.9
+PSA	84.4	81.2	87.5	78.9	84.1	81.3	87.4	79.0

### 3.6 Generalization ability Ttest of the ACP-tomato-seg model

To further evaluate the generalization ability and cross - dataset applicability of the ACP-Tomato -Seg model proposed in this paper, we conducted supplementary verification on the publicly available strawberry instance segmentation dataset (Pérez-Borrero et al., 2020). We identified four instance segmentation methods (Pérez-Borrero et al., 2020; Perez-Borrero et al., 2021; Cao et al., 2023; Guo et al., 2024) that had also been evaluated on this dataset as benchmarks, and carried out a comprehensive comparison using the same instance segmentation metrics (mAP, AP50, AP75, mAP_S_, mAP_M_, mAP_L_). The detailed comparison results are presented in **Table 7**. The results show that the method proposed in this paper significantly outperforms the selected comparative methods in the (AP50, mAP_S_, mAP_M_) metrics. In terms of the mAP, AP75, and mAP_L_ metrics, the performance of our model is comparable to that of StrawSeg. StrawSeg has a slight lead in these metrics (only 3.7%, 1.7%, and 3.1% higher respectively). Overall, the results of these comparative experiments on the Straw DI_Db1 dataset fully demonstrate that the effectiveness of our model design is not confined to a tomato dataset. The results of the comparative experiments on the public strawberry dataset strongly prove that the improved model proposed in this paper has good generalization ability and cross-dataset applicability. This indicates that the feature representations learned by the model and the structural optimizations are robust and can be effectively transferred to similar yet different fruit instance segmentation tasks, addressing concerns about generalization that may arise from testing only on self-built datasets.

**Table 7:** Performance comparison of our model and several existing models on the straw DI_Db1 test set.

Methods	mAP	AP50	AP75	mAP_S_	mAP_M_	mAP_L_
Pérez-Borrero et al. (2020)	43.8	74.2	45.1	7.5	51.7	75.9
Perez-Borrero et al. (2021)	52.6	69.4	57.8	17	65.3	53.3
StrawSeg (Cao et al., 2023)	80.0	89.8	83.8	40.9	83.3	97.1
StrawSnake (Guo et al., 2024)	59.23	81.54	66.73	24.26	71.29	82.87
Our	76.3	93.4	82.1	60.5	85.0	94.0

## 4 Discussion

This study successfully developed an instance segmentation-based tomato maturity detection and fruit segmentation method, ACP-Tomato-Seg, aiming to solve the technical challenges of precise tomato picking in complex field environments. In response to the deficiency of traditional object detection methods in obtaining fine contour information, the proposed ACP-Tomato-Seg method significantly enhances the model’s feature expression ability for tomatoes of different maturities, sizes, shapes, and with occlusion by innovatively introducing three modules: ADFRM, CMPRD, and PSA. Experimental results show that on the Tomato-Seg dataset, our method achieves excellent performance under both bounding box and mask evaluation metrics, with high levels of accuracy, recall rate, and mAP metrics, fully verifying the effectiveness of ACP-Tomato-Seg in tomato maturity detection and fruit segmentation in complex field environments. More crucially, to further validate the effectiveness and generalization ability of our model design, we conducted additional comparative experiments on the publicly available StrawDI_Db1 dataset (details can be found in **Table 7**). The results of these experiments indicate that, even when confronted with different fruit types and data distributions, ACP-Tomato-Seg significantly outperforms existing methods in terms of performance, especially in key precision metrics. This not only addresses concerns about the potential limitations of a single dataset but also further confirms that the performance improvements brought about by our proposed innovative modules (ADFRM, CMPRD) possess good robustness and generalization potential across datasets. Compared with traditional object detection methods, the instance segmentation method can provide more detailed fruit contour information. This is crucial for precise robot picking because accurate contour information can help the robot system locate and grasp fruits more accurately, reduce damage during the picking process, and improve the efficiency and quality of picking. Therefore, the instance segmentation method proposed in this study provides important technical support for the realization of intelligence and precision in tomato-picking robots. One limitation of this study is that our current work mainly focuses on the development and verification of image processing and machine vision algorithms, lacking a direct comparative analysis of fruit maturity detection results with the physicochemical indicators of tomatoes. Although our method achieves satisfactory performance at the image level, effectively distinguishing tomatoes at different maturity stages and achieving precise fruit segmentation, we have not yet explored the quantitative relationship between the maturity levels classified by images and the actual physicochemical maturity. For example, we have failed to measure indicators such as soluble solids, firmness, and color parameters of tomatoes in different maturity categories, thus unable to verify the accuracy and reliability of our image detection results from the physicochemical level. We admit that adding verification of physicochemical indicators will enable a more comprehensive and in-depth evaluation of the effectiveness of our method and enhance the scientific rigor of the research conclusion. It should be noted that the main objective of this study is to explore and verify the potential of deep learning methods based on instance segmentation in solving the problems of tomato maturity detection and fruit segmentation in complex field environments. Therefore, our research focuses on the design of the model structure, algorithm optimization, and performance evaluation on image datasets. Although physicochemical analyses are crucial for a comprehensive verification of the effectiveness of the maturity detection method, it is beyond the scope and focus of this study. An important direction for future research will be to address the limitations of this study, conduct simultaneous measurement and analysis of physicochemical indicators of tomatoes, and correlate image segmentation and maturity detection results with physicochemical data. For example, future studies can collect images of tomatoes at different maturity stages and simultaneously measure indicators such as soluble solids, firmness, and color parameters to establish a quantitative relationship model between image features and physicochemical indicators. This will contribute to a deeper understanding of the intrinsic relationship between image features and tomato maturity and provide a more scientific basis for rapid and non-destructive maturity detection methods based on images. Additionally, as stated in the conclusion section, model lightweighting and integration with the robot vision system are also important directions for our future research. We will explore model compression and acceleration techniques to reduce the number of model parameters and computational costs, enabling its deployment on mobile or embedded devices with limited computing resources. At the same time, we will strive to combine the ACP-Tomato-Seg method with the robot vision system to develop a prototype system of an automated tomato-picking robot and verify the application potential of this method in actual agricultural production.

## 5 Conclusions and future work

In order to solve the difficult problem of tomato ripenness detection and fruit segmentation in complex field environment, this paper pays special attention to the limitation that traditional target detection methods cannot meet the requirements of robot accurate picking for fruit contour information, and proposes an ACP-Tomato-Seg instance segmentation method. Through the proposed AOFRM, CMPRD and introduced PSA three innovative modules, the method effectively improved the feature expression ability of the model for tomatoes of different sizes, shapes and occlusion degrees, and realized accurate identification of tomato maturity and fine segmentation of fruit contour. AOFRM module uses adaptive receptive field and multi-directional feature extraction to effectively solve the segmentation problems caused by tomato occlusion and overlap. The CMPRD module improved the model’s ability to recognize tomatoes of different sizes and maturity levels through multi-scale feature fusion. By capturing the global context information, PSA module further enhances the accuracy of the model to extract details such as tomato contour and texture. The design of these modules cooperates with each other to improve the detection and segmentation performance of the model. The AOFRM module enhances the perception ability of the model to the tomato target, and the CMPRD module extracts multi-scale information to enable the model to understand the characteristics of the tomato target more comprehensively. The PSA module uses the multi-scale features extracted by CMPRD to guide the model to focus on important features through the attention mechanism, thereby improving the detection and segmentation accuracy of the model. Experimental results on Tomato-Seg dataset with six categories show that the proposed method achieves remarkable performance improvement. Under the Box task, the accuracy, recall rate, mAP50 and MAP50-95 reached 85.8%, 82.2%, 91.3% and 84.5% respectively. Under the Mask task, the accuracy, recall rate, mAP50 and MAP50-95 reached 85.5%, 82.1%, 91.2% and 83.3% respectively. To verify the generalization ability of the model, we also conducted comparative experiments on the StrawDI_Db1 dataset. Even when faced with different fruit types and data distributions, our method still significantly outperforms existing benchmark methods in the main evaluation metrics. This strongly supports the effectiveness of our model design and its robustness across datasets.

This study provides an effective technical solution for realizing accurate picking of tomatoes, but the number of model parameters and calculation is still large, which limits its deployment and application on lightweight equipment. The future work will focus on the following aspects: First, in order to verify the accuracy of maturity detection more scientifically, we will conduct simultaneous measurement and correlation analysis of physicochemical indicators. Secondly, the performance and robustness of the model will be tested in real field environments and on robot platforms, and optimizations will be made for uncontrolled conditions. At the same time, efforts will be devoted to the lightweighting of the model to enable its deployment on devices with limited resources. In addition, in-depth exploration of the integration of the model with the robot vision system will be carried out to develop an automated picking prototype system. Finally, it is planned to expand the diversity of the dataset to enhance the generalization ability of the model. In conclusion, future research will be dedicated to promoting the ACP-Tomato-Seg method from laboratory research to practical application and contributing to the development of smart agriculture.

## Data Availability

The original contributions presented in the study are included in the article/supplementary material. Further inquiries can be directed to the corresponding authors.

## References

[B1] Ang G. Zhiwei T. Wei M. Yuepeng S. Longlong R. Yuliang F.. (2024). Fruits hidden by green: an improved YOLOV8n for detection of young citrus in lush citrus trees. Front. Plant Sci. 15. doi: 10.3389/fpls.2024.1375118 PMC11039839 38660450

[B2] Appe S. N. Arulselvi G. Balaji G. N. (2023). CAM-YOLO: tomato detection and classification based on improved YOLOv5 using combining attention mechanism. PeerJ. Comput. Sci. 9. doi: 10.7717/peerj-cs.1463 PMC10403160 37547387

[B3] Arnal Barbedo J. G. (2019). Plant disease identification from individual lesions and spots using deep learning. Biosyst. Eng. 180, 96–107. doi: 10.1016/j.biosystemseng.2019.02.002

[B4] Cao L. Chen Y. Jin Q. (2023). Lightweight strawberry instance segmentation on low-power devices for picking robots. Electron. (Switzerland). 12. doi: 10.3390/electronics12143145

[B5] Chen C. Li B. Liu J. Bao T. Ren N. (2020). Monocular positioning of sweet peppers: An instance segmentation approach for harvest robots. Biosyst. Eng. 196, 15–28. doi: 10.1016/j.biosystemseng.2020.05.005

[B6] Cheng B. Misra I. Schwing A. G. Kirillov A. Girdhar R. (2021). “Masked-attention mask transformer for universal image segmentation,” in 2022 IEEE/CVF Conference on Computer Vision and Pattern Recognition (CVPR), New Orleans, LA, USA, 1280–1289. Available at: https://api.semanticscholar.org/CorpusID:244799297 (Accessed April 25, 2025).

[B7] Ding X. Guo Y. Ding G. Han J. (2019). “ACNet: Strengthening the Kernel Skeletons for Powerful CNN via Asymmetric Convolution Blocks,” in 2019 IEEE/CVF International Conference on Computer Vision (ICCV), Seoul, Korea (South). 1911–1920. Available online at: https://api.semanticscholar.org/CorpusID:199543841 (Accessed April 25, 2025).

[B8] Gao S.-H. Cheng M.-M. Zhao K. Zhang X.-Y. Yang M.-H. Torr P. (2021). Res2Net: A new multi-scale backbone architecture. IEEE Trans. Pattern Anal. Mach. Intell. 43, 652–662. doi: 10.1109/TPAMI.2019.2938758 31484108

[B9] Gongal A. Amatya S. Karkee M. Zhang Q. Lewis K. (2015). Sensors and systems for fruit detection and localization: A review. Comput. Electron. Agric. 116, 8–19. doi: 10.1016/j.compag.2015.05.021

[B10] Guo Z. Hu X. Zhao B. Wang H. Ma X. (2024). StrawSnake: A real-time strawberry instance segmentation network based on the contour learning approach. Electron. (Switzerland). 13. doi: 10.3390/electronics13163103

[B11] Guo J. Yang Y. Lin X. Memon M. S. Liu W. Zhang M.. (2023). Revolutionizing agriculture: real-time ripe tomato detection with the enhanced tomato-YOLOv7 system. IEEE Access 11, 133086–133098. doi: 10.1109/ACCESS.2023.3336562

[B12] He K. Gkioxari G. Dollár P. Girshick R. (2017). “Mask R-CNN,” in 2017 IEEE International Conference on Computer Vision (ICCV). (Venice, Italy: IEEE), 2980–2988. doi: 10.1109/ICCV.2017.322

[B13] He K. Zhang X. Ren S. Sun J. (2014). Spatial pyramid pooling in deep convolutional networks for visual recognition. IEEE Trans. Pattern Anal. Mach. Intell. 37, 1904–1916. doi: 10.1109/TPAMI.2015.2389824 26353135

[B14] Hou Q. Zhou D. Feng J. (2021). “Coordinate attention for efficient mobile network design,” in 2021 IEEE/CVF Conference on Computer Vision and Pattern Recognition (CVPR), Nashville, TN, USA. 13708–13717. Available online at: https://api.semanticscholar.org/CorpusID:232110359 (Accessed April 25, 2025).

[B15] Howard A. G. Zhu M. Chen B. Kalenichenko D. Wang W. Weyand T.. (2017). “MobileNets: efficient convolutional neural networks for mobile vision applications,” in ArXiv, Ithaca, New York, USA. Available online at: https://api.semanticscholar.org/CorpusID:12670695. abs/1704.04861 (Accessed April 25, 2025).

[B16] Hu J. Shen L. Albanie S. Sun G. Wu E. (2017). “Squeeze-and-Excitation networks,” in 2018 IEEE/CVF Conference on Computer Vision and Pattern Recognition, Salt Lake City, UT, USA. 7132–7141. Available at: https://api.semanticscholar.org/CorpusID:140309863 (Accessed April 25, 2025).

[B17] Jocher G. (2020). YOLOv5 by ultralytics. (Geneva, Switzerland: Zenodo). doi: 10.5281/zenodo.3908559

[B18] Jocher G. Chaurasia A. Qiu J. (2023). Ultralytics YOLO. Available online at: https://github.com/ultralytics/ultralytics (Accessed April 25, 2025).

[B19] Li C. Li L. Jiang H. Weng K. Geng Y. Li L.. (2022). “YOLOv6: A single-stage object detection framework for industrial applications,” in ArXiv, Ithaca, New York, USA. Available online at: https://api.semanticscholar.org/CorpusID:252110986. abs/2209.02976 (Accessed April 25, 2025).

[B20] Li Y. Liao J. Wang J. Luo Y. Lan Y. (2023). Prototype network for predicting occluded picking position based on lychee phenotypic features. Agronomy 13. doi: 10.3390/agronomy13092435

[B21] Liu Y. Zheng H. Zhang Y. Zhang Q. Chen H. Xu X.. (2023). Is this blueberry ripe?”: a blueberry ripeness detection algorithm for use on picking robots. Front. Plant Sci. 14. doi: 10.3389/fpls.2023.1198650 PMC10289036 37360727

[B22] Lv W. Xu S. Zhao Y. Wang G. Wei J. Cui C.. (2023). “DETRs beat YOLOs on real-time object detection,” in 2024 IEEE/CVF Conference on Computer Vision and Pattern Recognition (CVPR), Seattle, WA, USA. 16965–16974. Available online at: 10.1109/CVPR52733.2024.01605 (Accessed April 25, 2025).

[B23] Ma M. Taylor P. W. J. Chen D. Vaghefi N. He J.-Z. (2023). Major soilborne pathogens of field processing tomatoes and management strategies. Microorganisms 11. doi: 10.3390/microorganisms11020263 PMC9958975 36838227

[B24] Nguyen D.-L. Vo X.-T. P A. C J. J K.-H. (2024). “Improved tomato detector supporting for automatic harvesting systems,” in the 13th Conference on Information Technology and Its Applications. eds. C.-P. N. T. T. L.-K. N.-A. N. Q.-V. Nguyen N. T. Huynh (Cham: Springer Nature Switzerland), 348–359.

[B25] Oliveira L. F. P. Moreira A. P. Silva M. F. (2021). Advances in agriculture robotics: A state-of-the-art review and challenges ahead. Robotics 10. doi: 10.3390/robotics10020052

[B26] Pérez-Borrero I. Marín-Santos D. Gegúndez-Arias M. E. Cortés-Ancos E. (2020). A fast and accurate deep learning method for strawberry instance segmentation. Comput. Electron. Agric. 178, 105736. doi: 10.1016/j.compag.2020.105736

[B27] Perez-Borrero I. Marin-Santos D. Vasallo-Vazquez M. J. Gegundez-Arias M. E. (2021). A new deep-learning strawberry instance segmentation methodology based on a fully convolutional neural network. Neural Comput. Appl. 33, 15059–15071. doi: 10.1007/s00521-021-06131-2

[B28] Redmon J. Farhadi A. (2018). “YOLOv3: an incremental improvement,” in ArXiv, Ithaca, New York, USA. Available at: https://api.semanticscholar.org/CorpusID:4714433. abs/1804.02767 (Accessed April 25, 2025).

[B29] Septiarini A. Sunyoto A. Hamdani H. Kasim A. A. Utaminingrum F. Hatta H. R. (2021). Machine vision for the maturity classification of oil palm fresh fruit bunches based on color and texture features. Sci. Hortic. 286, 110245. doi: 10.1016/j.scienta.2021.110245

[B30] Tang Y. Chen M. Wang C. Luo L. Li J. Lian G.. (2020). Recognition and localization methods for vision-based fruit picking robots: A review. Front. Plant Sci. 11. doi: 10.3389/fpls.2020.00510 PMC7250149 32508853

[B31] Tian Y. Ye Q. Doermann D. (2025). “YOLOvl2: Attention-Centric Real-Time Object,” in ArXiv, Ithaca, New York, USA. Available online at: https://arxiv.org/abs/2502.12524 (Accessed April 25, 2025).

[B32] Tsironis V. Bourou S. Stentoumis C. (2020). “Tomatod: Evaluation of object detection algorithms on a new real-world tomato dataset,” in International Archives of the Photogrammetry, Remote Sensing and Spatial Information Sciences - ISPRS Archives (ISPRS Archives International Society for Photogrammetry and Remote Sensing), 1077–1084. doi: 10.5194/isprs-archives-XLIII-B3-2020-1077-2020

[B33] Vaswani A. Shazeer N. M. Parmar N. Uszkoreit J. Jones L. Gomez A. N.. (2017). “Attention is All you Need,” in Neural information Processing Systems, in NIPS’17. (Red Hook, NY, USA Curran Associates Inc.)., pp. 6000–6010. Available online at: https://api.semanticscholar.org/CorpusID:13756489 (Accessed April 25, 2025).

[B34] Wan P. Toudeshki A. Tan H. Ehsani R. (2018). A methodology for fresh tomato maturity detection using computer vision. Comput. Electron. Agric. 146, 43–50. doi: 10.1016/j.compag.2018.01.011

[B35] Wang C.-Y. Bochkovskiy A. Liao H.-Y. M. (2022). “YOLOv7: trainable bag-of-freebies sets new state-of-the-art for real-time object detectors,” in 2023 IEEE/CVF Conference on Computer Vision and Pattern Recognition (CVPR), Vancouver, BC, Canada. 7464–7475. Available online at: https://api.semanticscholar.org/CorpusID:250311206.

[B36] Wang A. Chen H. Liu L. Chen K. Lin Z. Han J.. (2024). “YOLOv10: real-time end-to-end object detection,” in ArXiv, Ithaca, New York, USA. Available at: https://api.semanticscholar.org/CorpusID:269983404. abs/2405.14458 (Accessed April 25, 2025).

[B37] Wang T. Chen B. Zhang Z. Li H. Zhang M. (2022). Applications of machine vision in agricultural robot navigation: A review. Comput. Electron. Agric. 198, 107085. doi: 10.1016/j.compag.2022.107085

[B38] Wang C. Wang C. Wang L. Wang J. Liao J. Li Y.. (2023). A lightweight cherry tomato maturity real-time detection algorithm based on improved YOLOV5n. Agronomy 13. doi: 10.3390/agronomy13082106

[B39] Wang Q. Wu B. Zhu P. F. Li P. Zuo W. Hu Q. (2019). “ECA-net: efficient channel attention for deep convolutional neural networks,” in 2020 IEEE/CVF Conference on Computer Vision and Pattern Recognition (CVPR), Seattle, WA, USA. 11531–11539. Available online at: https://api.semanticscholar.org/CorpusID:203902337 (Accessed April 25, 2025).

[B40] Wang C.-Y. Yeh I.-H. Liao H. (2024). YOLOv9: learning what you want to learn using programmable gradient information. ArXiv. Available online at: https://api.semanticscholar.org/CorpusID:267770251. abs/2402.13616 (Accessed April 25, 2025).

[B41] Woo S. Park J. Lee J.-Y. Kweon I.-S. (2018). “CBAM: convolutional block attention module,” in ArXiv, Ithaca, New York, USA. Available online at: https://api.semanticscholar.org/CopusID:49867180. abs/1807.06521 (Accessed April 25, 2025).

[B42] Yu F. Koltun V. (2015). “Multi-scale context aggregation by dilated convolutions,” in ArXiv, Ithaca, New York, USA. Available online at: https://api.semanticscholar.org/CorpusID:17127188. abs/1511.07122 (Accessed April 25, 2025).

[B43] Zhang H. Tang C. Sun X. Fu L. (2023). A refined apple binocular positioning method with segmentation-based deep learning for robotic picking. Agronomy 13. doi: 10.3390/agronomy13061469

[B44] Zhu X. Hu H. Lin S. Dai J. (2018). “Deformable convNets V2: more deformable, better results,” in 2019 IEEE/CVF Conference on Computer Vision and Pattern Recognition (CVPR), Long Beach, CA, USA. 9300–9308. Available online at: https://api.semanticscholar.org/CorpusID:53745820 (Accessed April 25, 2025).

